# Supplementary figures and images for: Glycosylation of Stilbene Compounds by Cultured Plant Cells
Source: Molecules. 2020 Mar 22;25(6):1437. doi: 10.3390/molecules25061437 (PMC7145310; doi:10.3390/molecules25061437)

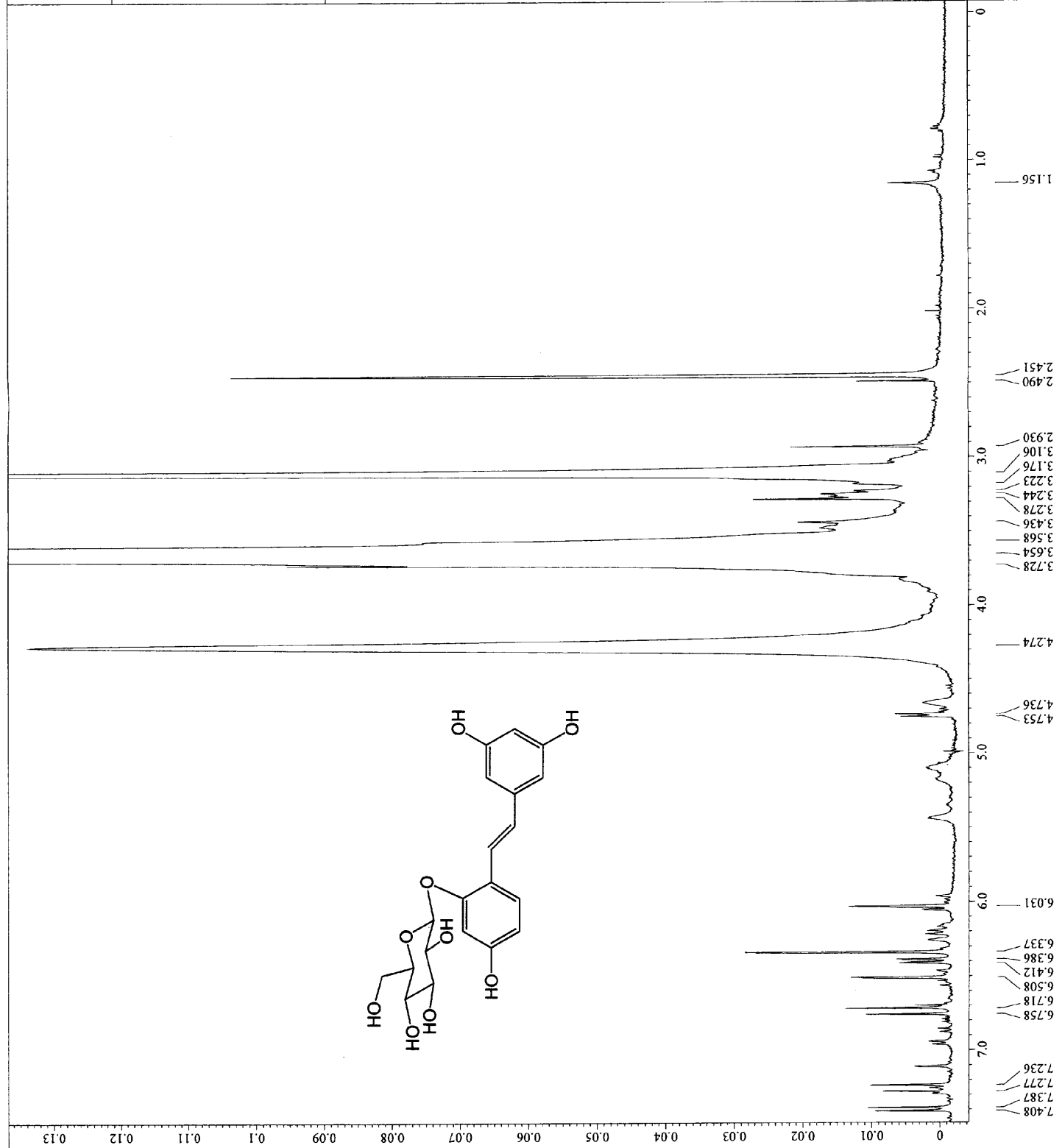

Supplement: Supplementary file 1 [file molecules-25-01437-s001.zip › Figure S2. 1H NMR of oxyresveratrol 2-beta-glucoside (87% purity).pdf]

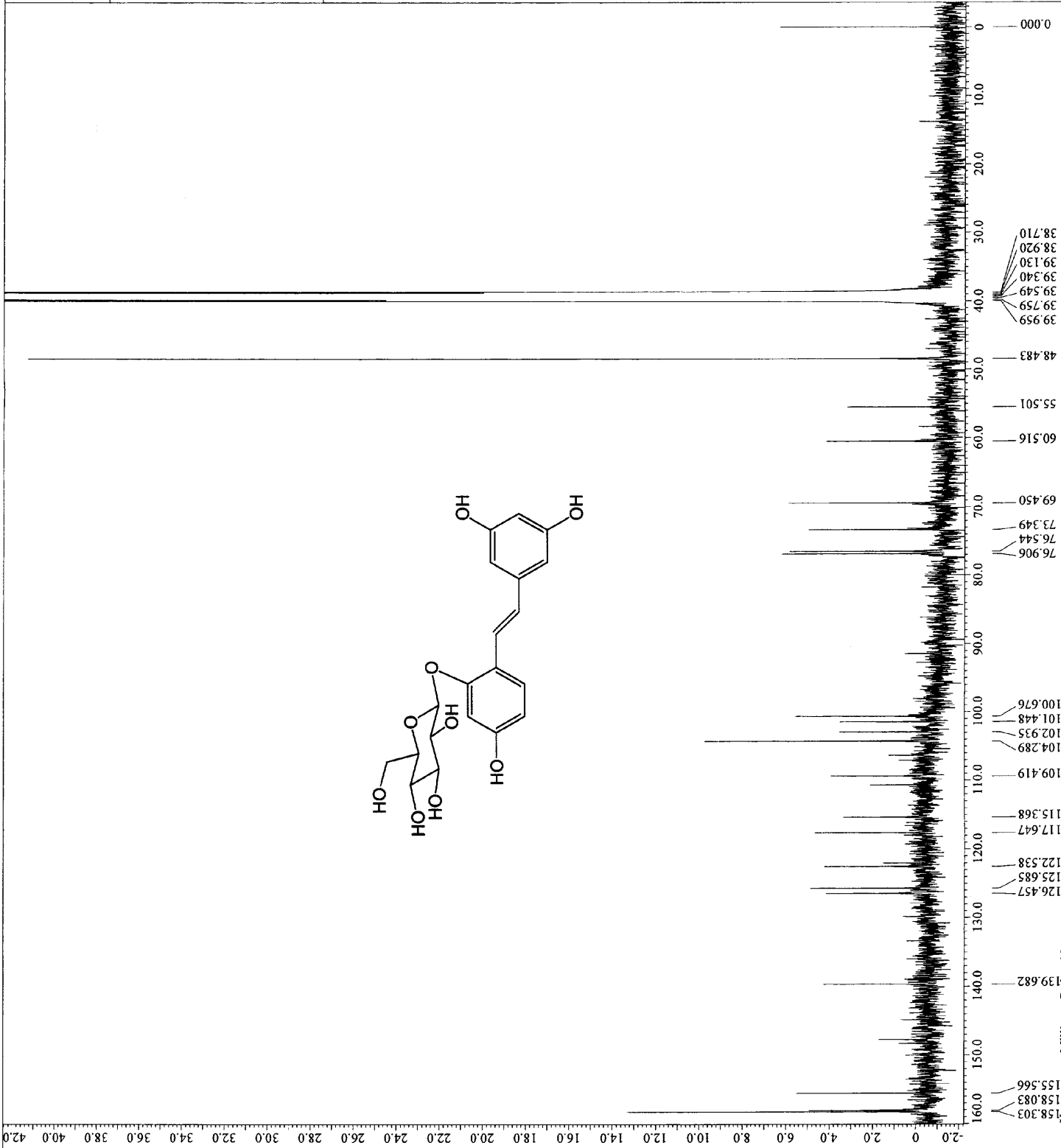

Supplement: Supplementary file 1 [file molecules-25-01437-s001.zip › Figure S3. 13C NMR of oxyresveratrol 2-beta-glucoside (87% purity).pdf]

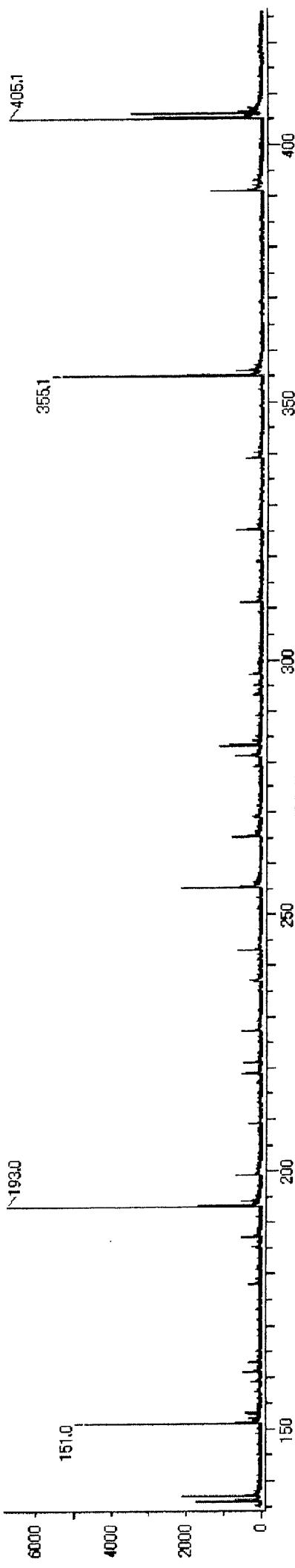

Supplement: Supplementary file 1 [file molecules-25-01437-s001.zip › Figure S1. ESIMS of oxyresveratrol 2-beta-glucoside.pdf]

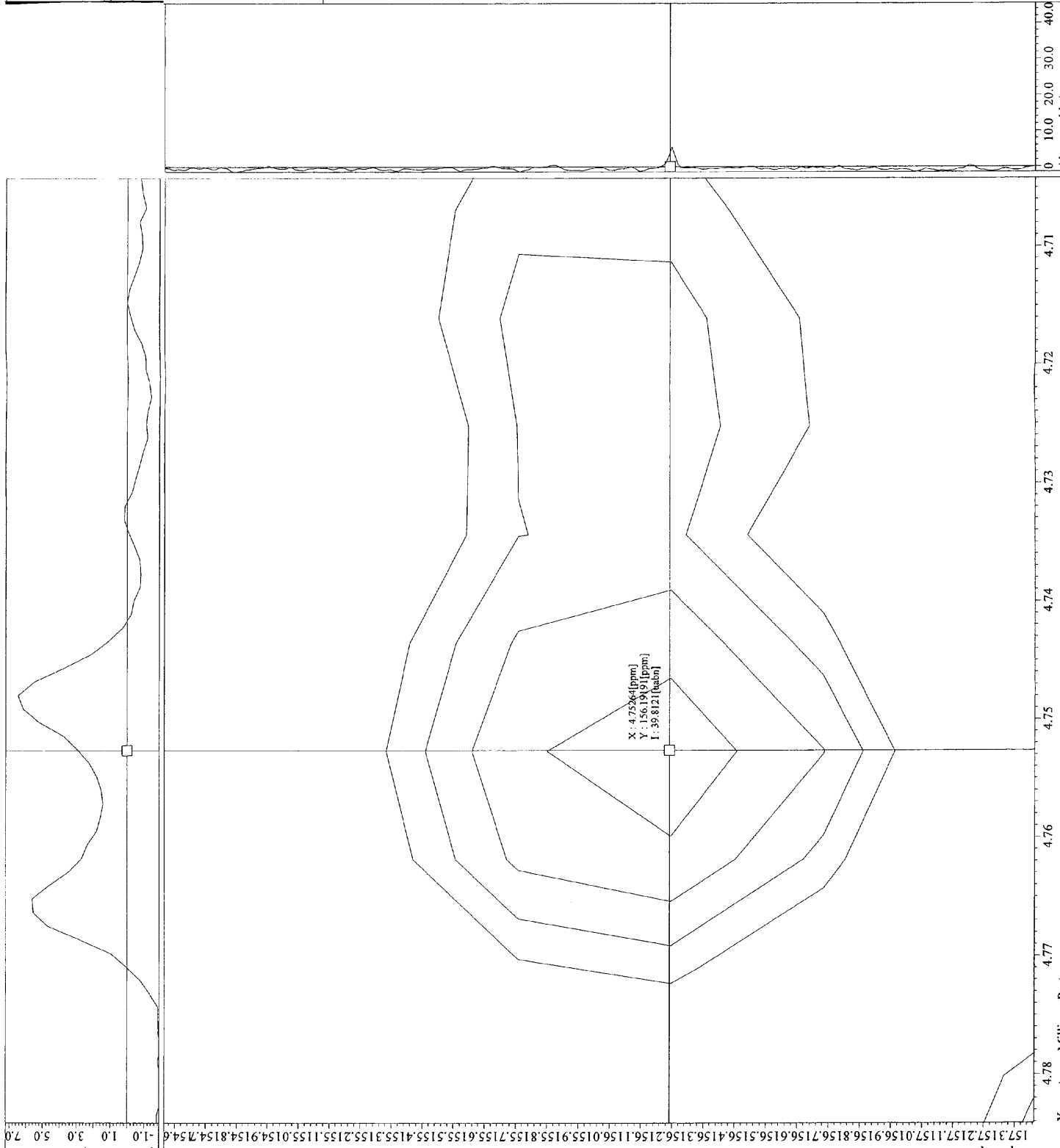

Supplement: Supplementary file 1 [file molecules-25-01437-s001.zip › Figure S5. HMBC of oxyresveratrol 2-beta-glucoside (87% purity) (enlarged).pdf]

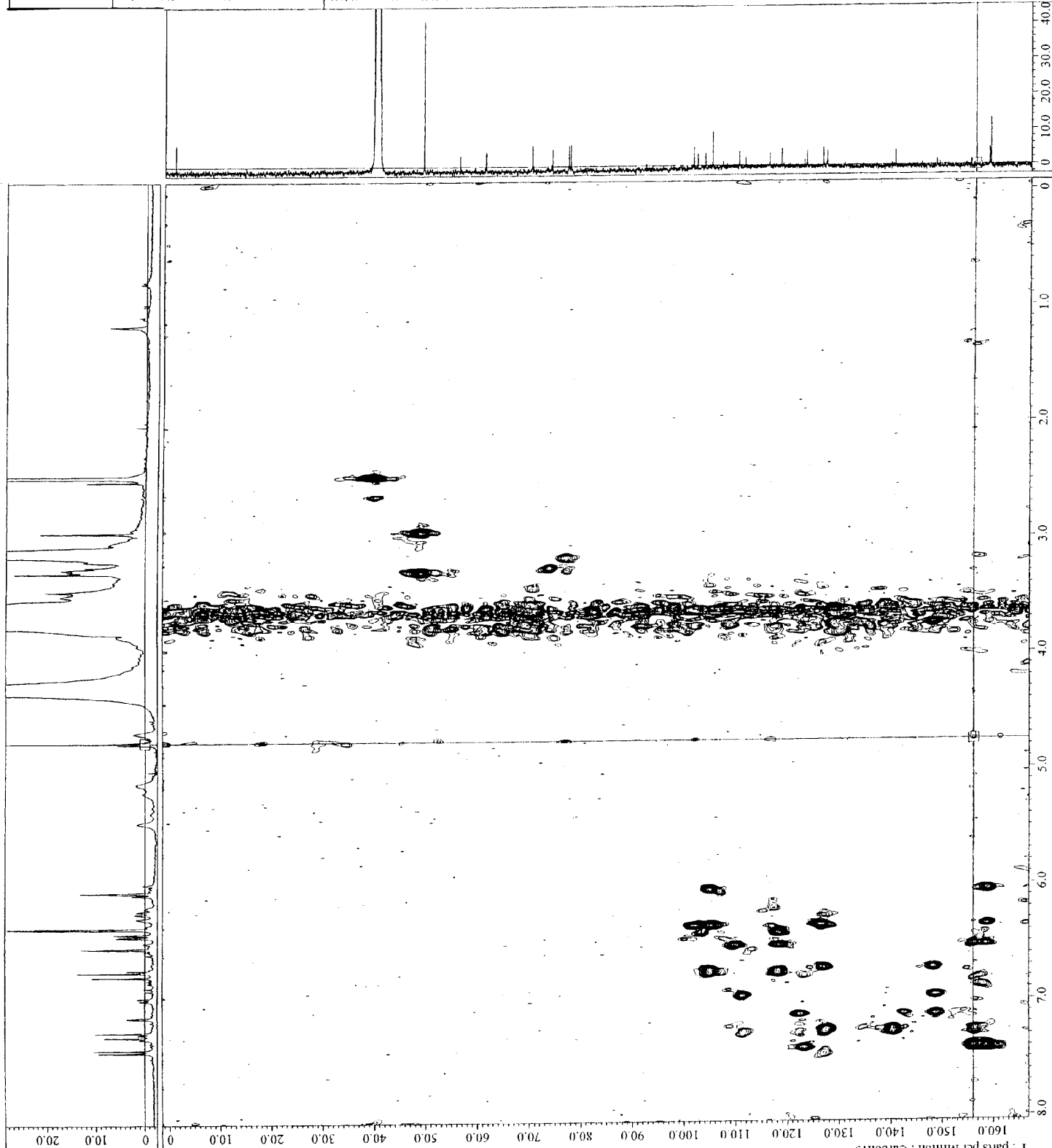

Supplement: Supplementary file 1 [file molecules-25-01437-s001.zip › Figure S4. HMBC of oxyresveratrol 2-beta-glucoside (87% purity).pdf]

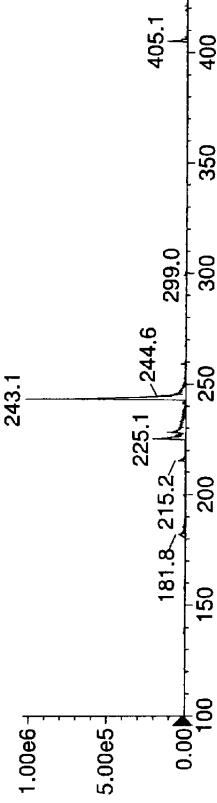

Supplement: Supplementary file 1 [file molecules-25-01437-s001.zip › Figure S6. ESIMS of gnetol 2-beta-glucoside.pdf]

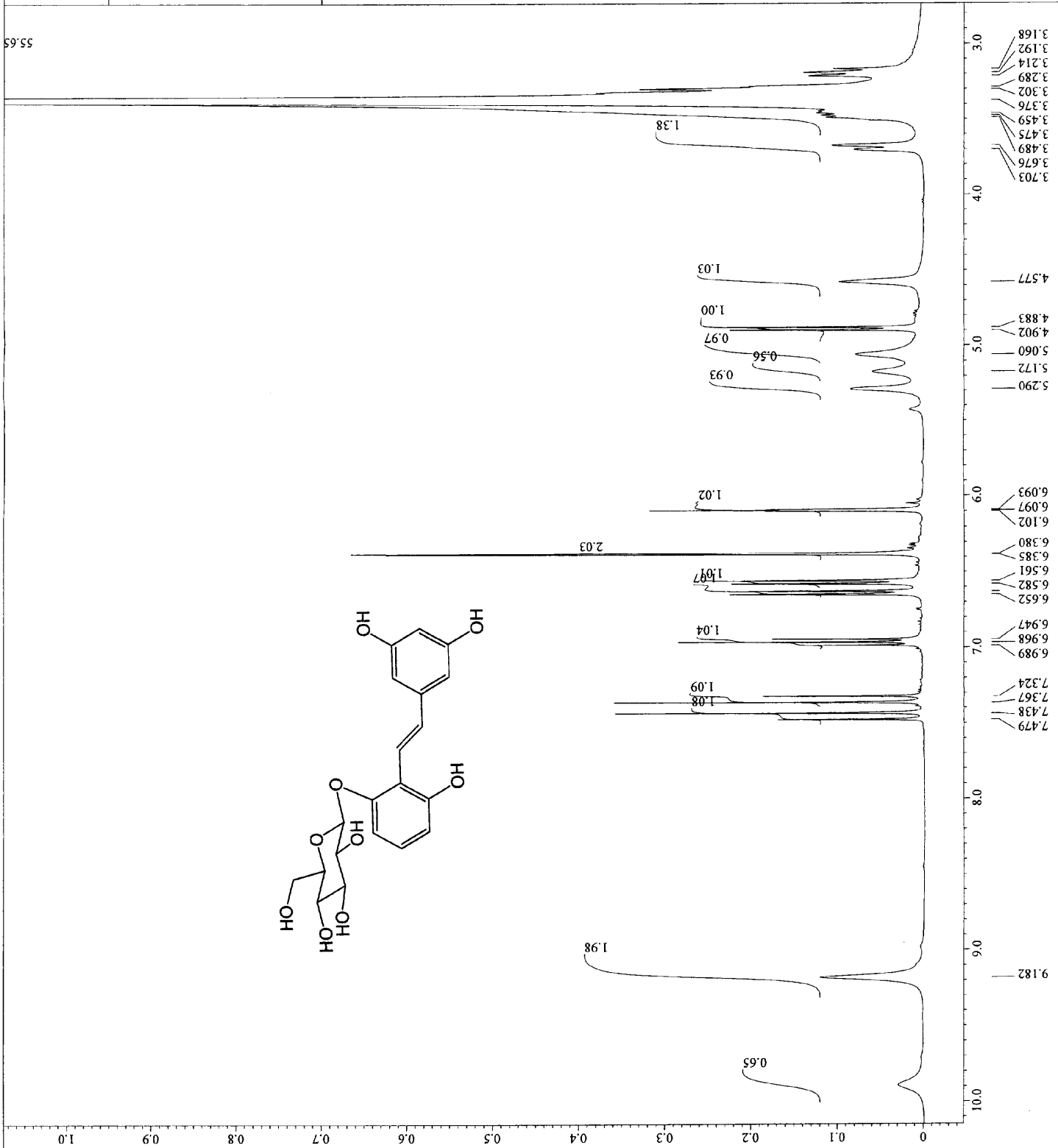

Supplement: Supplementary file 1 [file molecules-25-01437-s001.zip › Figure S7. 1H NMR of gnetol 2-beta-glucoside (95% purity).pdf]

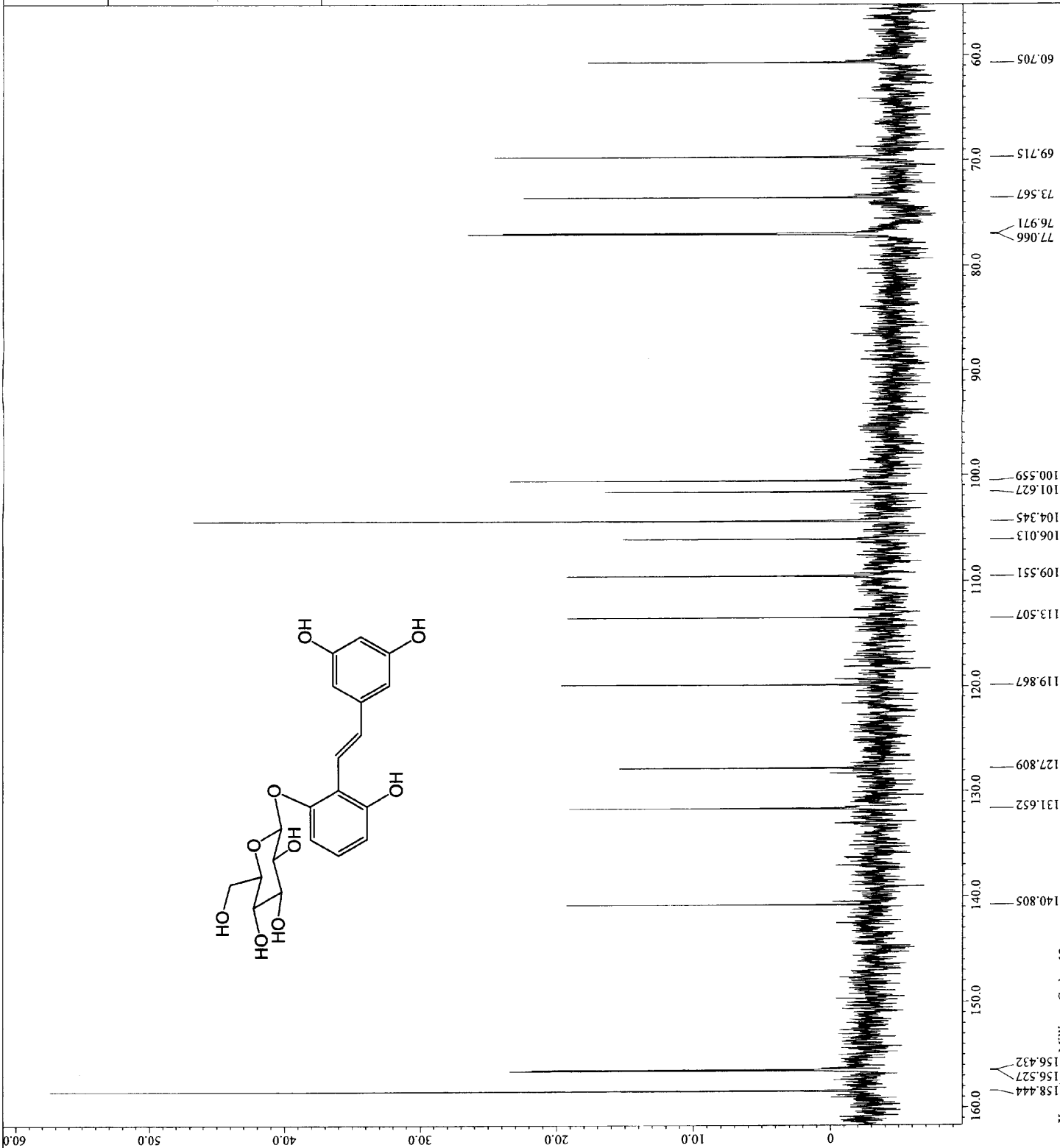

Supplement: Supplementary file 1 [file molecules-25-01437-s001.zip › Figure S8. 13C NMR of gnetol 2-beta-glucoside (95% purity).pdf]

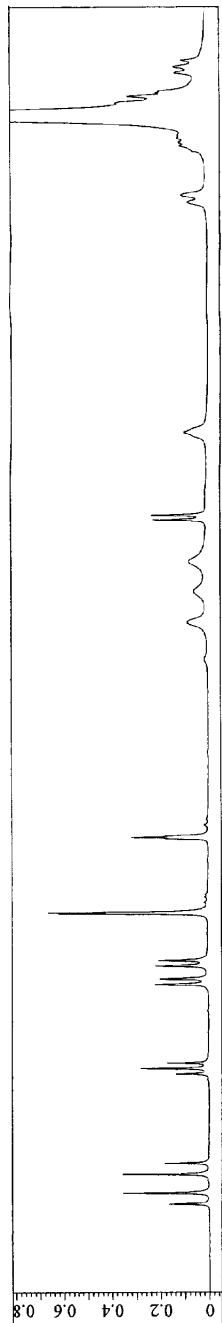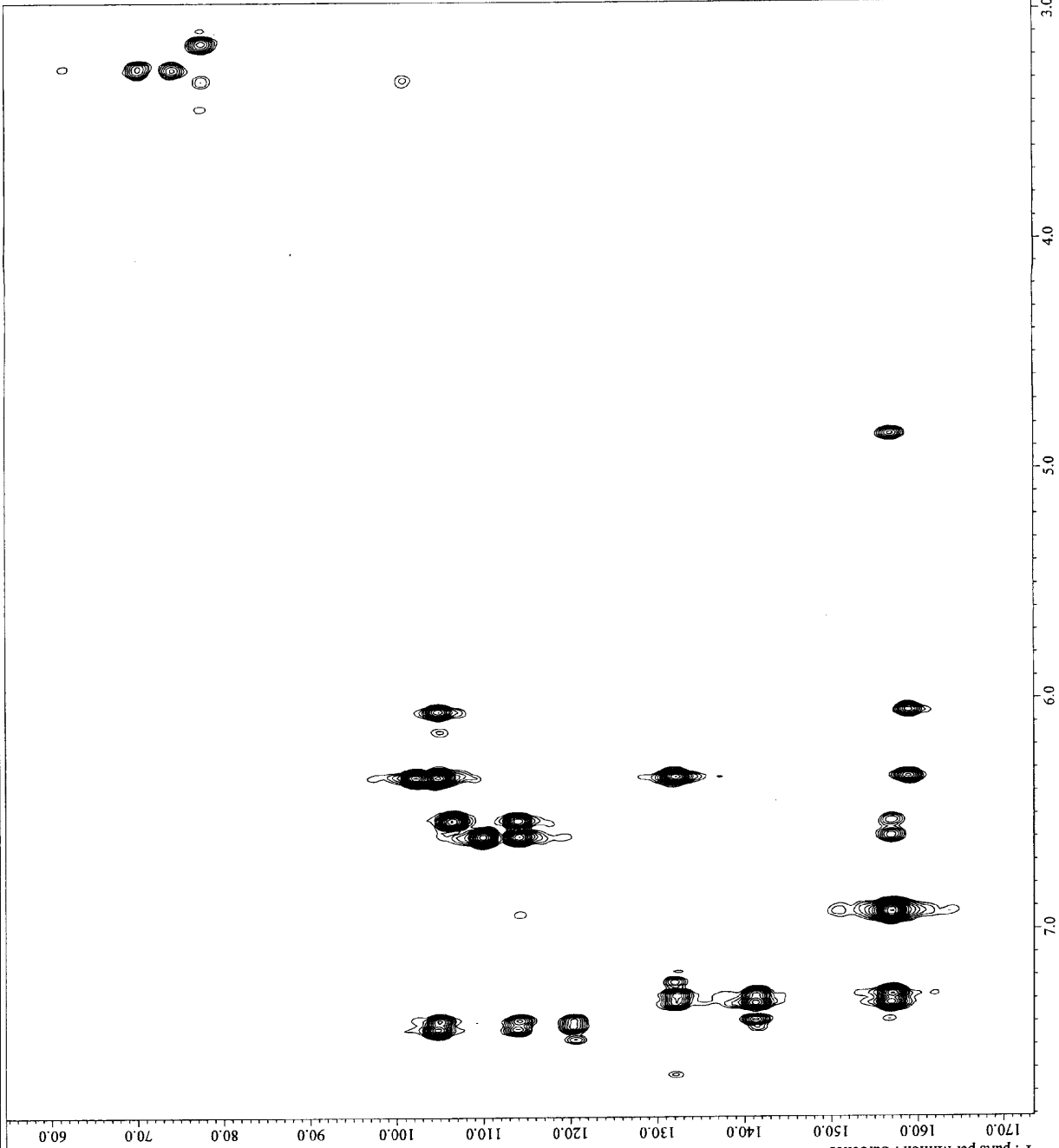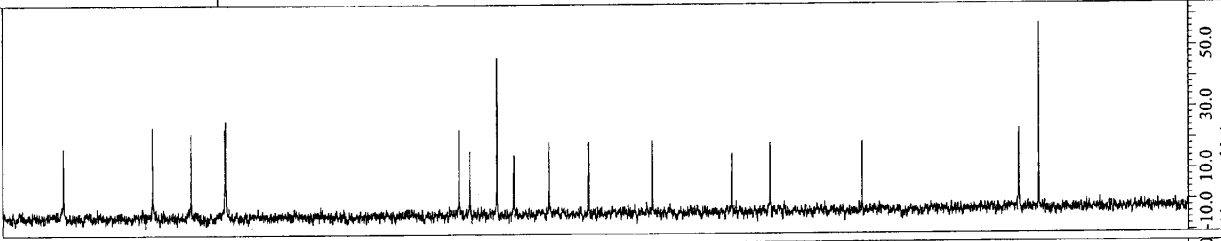

Supplement: Supplementary file 1 [file molecules-25-01437-s001.zip › Figure S9. HMBC of gnetol 2-beta-glucoside (95% purity).pdf]
